# Identification of vaccine-derived rotavirus strains in children with acute gastroenteritis in Japan, 2012-2015

**DOI:** 10.1371/journal.pone.0184067

**Published:** 2017-09-13

**Authors:** Mei Kaneko, Sayaka Takanashi, Aksara Thongprachum, Nozomu Hanaoka, Tsuguto Fujimoto, Koo Nagasawa, Hirokazu Kimura, Shoko Okitsu, Masashi Mizuguchi, Hiroshi Ushijima

**Affiliations:** 1 Department of Developmental Medical Sciences, Graduate School of Medicine, The University of Tokyo, Tokyo, Japan; 2 Division of Microbiology, Department of Pathology and Microbiology, School of Medicine, Nihon University, Tokyo, Japan; 3 Infectious Disease Surveillance Center, National Institute of Infectious Diseases, Tokyo, Japan; University of Hong Kong, HONG KONG

## Abstract

Two live attenuated oral rotavirus vaccines, Rotarix and RotaTeq, have been introduced as voluntary vaccination in Japan since 2011 and 2012, respectively. Effectiveness of the vaccines has been confirmed, whereas concerns such as shedding of the vaccine strains and gastroenteritis cases caused by vaccine strains are not well assessed. We aimed to identify the vaccine strains in children with acute gastroenteritis (AGE) to investigate the prevalence of AGE caused by vaccination or horizontal transmission of vaccine strains. A total of 1,824 stool samples were collected from children with AGE at six outpatient clinics in 2012–2015. Among all, 372 group A rotavirus (RVA) positive samples were screened for vaccine components by real-time RT-PCR which were designed to differentiate vaccine strains from rotavirus wild-type strains with high specificity. For samples possessing both vaccine and wild-type strains, analyses by next-generation sequencing (NGS) were conducted to characterize viruses existed in the intestine. As a result, Rotarix-derived strains were identified in 6 of 372 (1.6%) RVA positive samples whereas no RotaTeq strain was detected. Among six samples, four possessed Rotarix-derived strains while two possessed both Rotarix-derived strains and wild-type strains. In addition, other pathogens such as norovirus, enterovirus and *E*.*coli* were detected in four samples. The contribution of these vaccine strains to each patient’s symptoms was unclear as all of the cases were vaccinated 2–14 days before sample collection. Proportion of average coverage for each segmented gene by NGS strongly suggested the concurrent infection of the vaccine-derived strain and the wild-type strain rather than reassortment of these two strains in one sample. This is the first study to report the prevalence of vaccine-derived strains in patients with RVA AGE in Japan as 1.6% without evidence of horizontal transmission. The results emphasized the importance of continuous monitoring on vaccine strains and their clinical impacts on children.

## Introduction

Rotavirus infection is a leading cause of acute gastroenteritis (AGE) among children under five years of age causing approximately 215,000 deaths in a year worldwide [1]. Rotaviruses are nonenveloped triple layered viruses which contain 11 segments of double-stranded RNA coding for six structural proteins (VP1-VP4, VP6 and VP7) and six nonstructural proteins (NSP1-NSP5/6) (2). Group A rotaviruses (RVA) are a leading cause of AGE in children and are further classified based on genotypes of two outer capsid proteins, VP7 (G-type) and VP4 (P-type) [2]. In addition to G/P-genotyping, a complete genome classification system was developed by rotavirus classification working group (RCWG) [3]. The VP7-VP4-VP6-VP1-VP2-VP3-NSP1-NSP2-NSP3-NSP4-NSP5/6 genes of each strain were described as Gx-Px-Ix-Rx-Cx-Mx-Ax-Nx-Tx-Ex-Hx (x = Arabic numbers starting from one, representing each genotype) to represent a genotype constellation. Since RNA segments from different rotaviruses within the same group would reassort with high frequency during dual infections of cells, a comprehensive classification on 11 genome segments allows us to identify reassortments to confirm interspecies transmission [2,4,5].

Two live attenuated oral rotavirus vaccines, Rotarix (GlaxoSmithKine, Rixensart, Belgium) and RotaTeq (Merck & Co., Whitehouse Station, NJ, USA) were first licensed in 2004 in Mexico and the Dominican Republic, and in 2006 in the United States (US), respectively. Rotarix is a two-dose monovalent human rotavirus vaccine while RotaTeq is a three-dose pentavalent bovine-human reassortant vaccine [6,7]. Eighty six countries have introduced them into their national immunization programs since WHO recommended routine immunization of rotavirus vaccines to all infants in 2009 [8,9]. Both vaccines were found to be highly effective against severe rotavirus gastroenteritis and rotavirus-associated hospitalization in early vaccine-introducing countries such as the US, Mexico, Belgium and Australia [10–14]. Following vaccine introduction in 2006 (RotaTeq) and 2008 (Rotarix) in the US, the effectiveness for ≥1 dose of any rotavirus vaccine was 80% (95% CI, 74%–85%) against rotavirus hospitalizations and emergency department visits in 2009–2011 [10].

Despite the benefits of rotavirus vaccines, several concerns need to be mentioned. The first is shedding of vaccine strains. After the first dose of administration, the peak time of shedding occurred between day 4 and day 7, with positive detection rate of 80–90% by real-time RT-PCR [15]. For immunocompetent infants, the shedding was detected as early as one day and as late as 28 days after the administration. The duration of shedding is prolonged in immunocompromised infants, for instance, for more than six months in infants with severe combined immune deficiency (SCID) [16,17]. The second is AGE caused by vaccine strains or vaccine-derived reassortants. Some cases resulted from administration of the vaccines while others from horizontal transmissions of vaccine strains. According to a previous study in the US, vaccine or vaccine-reassortant rotavirus strains were detected in stool samples from five of 106 (4.7%) immunocompetent children who required treatment for rotavirus gastroenteritis [18]. To provide the evidence of horizontal transmission of the vaccine strains, a study documented the occurrence of RotaTeq vaccine-derived rotavirus transmission from a vaccinated infant to his older unvaccinated sibling, resulting in symptomatic AGE which required emergency department care [19]. In addition, several studies have reported severe and chronic gastroenteritis caused by vaccine strains in children with SCID [17,20].

Japan is still in the initial stage of using rotavirus vaccines to control rotavirus infections among children. Rotarix and RotaTeq were introduced as voluntary vaccination in November 2011 and July 2012, respectively. The effectiveness of the vaccines has been confirmed since two vaccines were introduced with the average vaccine coverage being 47.6% between July 2012 and November 2014 [21–23]. However, the concerns as mentioned above remain to be clarified. Risk-benefit analyses on rotavirus vaccines are essential in the introduction of these two vaccines into the national immunization program. Therefore, the objective of this study is to investigate the prevalence of rotavirus vaccine derived strain in Japanese children with AGE to assess the risks of rotavirus vaccines by elucidating horizontal transmission of the vaccine strains.

## Materials and methods

### Study samples

A total of 1,824 stool samples from patients with AGE at six outpatient pediatric clinics in Hokkaido, Tokyo, Shizuoka, Kyoto, Osaka and Saga were collected from July 2012 to June 2015. The demographic characteristics and clinical symptoms were collected from medical records based on the factors described in a previous study [18]. Age of the patients ranged from neonate to 15 years (0–178 months; median age of 17 months). All stool samples were initially screened by a method of multiplex RT-PCR which was designed for detection of 11 diarrhea causing viruses including rotavirus (group A, B, and C), norovirus (GI and GII), adenovirus, astrovirus, sapovirus, aichivirus, parechovirus and enterovirus [24]. Additionally, stool samples were cultured and tested for bacteria when patient exhibited suspicious symptoms such as bloody stool.

### Viral RNA extraction

Viral RNA was extracted from 10% (w/v) stool suspension with carrier RNA using QIAamp Viral RNA mini kit (QIAGEN, Hilden, Germany) according to the manufacturer`s instruction. For next-generation sequencing (NGS), RNA was extracted using the same method but without adding carrier RNA.

### Real-time RT-PCR for detection of vaccine components

Rotarix NSP2 assay and RotaTeq VP6 assay are quantitative real-time RT-PCR assays for detection of Rotarix and RotaTeq vaccine components in stool samples [25]. The assays were designed for vaccine specific targets in genomes of Rotarix NSP2 gene (281 bp) and RotaTeq VP6 gene (100 bp), which were the appropriate targets to differentiate the vaccine strains from wild-type RVA strains. Rotavirus NSP3 assay was established for detection of wide range of clinically important rotaviruses [26] and was used to titrate wild-type strains in this study. All the assays were conducted using Applied Biosystems StepOnePlus Real-Time PCR System (Applied Biosystems, Foster city, CA, USA) in accordance with the procedures described in previous studies [25,26]. Primers and probes used in the assays are described in S1 Table.

### Quantitative real-time RT-PCR for other viral pathogens

Quantitative real-time RT-PCR for norovirus and enterovirus were carried out as previously described [27,28].

### Conventional RT-PCR and sanger sequencing

Reverse transcription was performed with denatured RNA template using SuperScript III Reverse Transcriptase (Invitrogen, Carlsbad, CA, USA) and random primer (Takara, Shiga, Japan) according to the manufacturer's instructions. For those samples that had difficulty in amplifying target genes in the downstream PCR, gene specific primers were used instead of random primer to increase the specificity and sensitivity in PCR (S2 Table).

PCR was performed to amplify whole or part of four rotavirus segmented genes: VP7, VP4, VP6 and NSP2. The primers used for each gene are shown in S2 Table. The reaction was carried out using GoTaq DNA Polymerase (Promega, Madison, WI, USA) in accordance with manufacturer's instructions and PCR conditions in the previous studies shown in S2 Table. For those samples that had difficulties in amplification, PCR was then performed using PrimeSTAR GXL DNA polymerase (Takara, Shiga, Japan) which had higher efficiency [29].

Sequencing was conducted for PCR amplicons using ABI Prism BigDye termination cycle sequencing reaction kit (Applied Biosystems, Foster, CA, USA) according to the manufacturer`s instruction. The sequence data were collected by an ABI Prism 310 Genetic Analyzer (Applied Biosystems, Foster city, CA, USA). Nucleotide alignments were edited and analyzed using MEGA 6 [30], BioEdit [31] and Basic Local Alignment Search Tool (BLAST) [32]. The obtained sequences were aligned and compared with the corresponding genes of Rotarix original strain (accession No.JX943614, JX943612, JX943613 and JX943605 for VP7, VP4, VP6 and NSP2 gene, respectively) [25].

### Analysis by NGS

RNA sequencing library was prepared using NEBNext Ultra RNA Library Prep Kit for Illumina (New England Biolabs, Ipswich, MA, USA). Nucleotide sequencing was carried out on Miseq II (Illumina, San Diego, CA, USA) using MiSeq Reagent Kit v3 to generate 300-cycle paired-end reads. To construct contigs from the obtained short reads, de novo assembling were conducted using CLC genomics workbench (QIAGEN, Hilden, Germany) and VirusTap [33]. Contigs derived from either rotavirus wild-type strains or Rotarix strain were identified by BLAST search. Genotypes of each contigs were determined by an automated genotyping tool RotaC 2.0 (http://Rotac.regatools.be/) according to the guideline proposed by RCWG.

### GenBank accession numbers

The representative gene sequences of RVA strains obtained in this study were deposited into GenBank under the accession numbers KY616885-KY616907 (S3 Table).

### Ethical clearance

Written informed consents were obtained from the guardians on behalf of the minors/children enrolled in our study. The study was approved by the ethical committees of The University of Tokyo and Nihon University under the registration number of 1139 and 25-13-0, respectively.

## Results

### Demographic characteristics and clinical symptoms of positive cases in Rotarix NSP2 assay

In total, 372 stool samples were positive for RVA and analyzed further in this study. Among them, partial NSP2 gene of Rotarix strain was detected in seven samples (1.9%), whereas no products of RotaTeq VP6 gene strain were detected. The demographic characteristics and clinical symptoms of seven positive cases in Rotarix NSP2 assay are summarized in Table 1. Except for patient No.3, all patients had been administered either 1^st^ dose or 2^nd^ dose of Rotarix and the samples were collected one to 14 days after the vaccinations. All cases exhibited mild and various combinations of AGE symptoms.

**Table 1:** Demographic characteristics and clinical symptoms of seven positive cases in Rotarix NSP2 assay.

Patient No.	Age (month)	Gender	Location	Vaccination history and date	Date of illness onset and sample collection	Diarrhea (number of episodes)	Vomiting (number of episodes)	Fever (°C)	Medical history	Sibling	Daycare attendance
**1**	3	Male	Shizuoka	Rotarix 1^st^ Dose 2013/1/16	2013/1/18 2013/1/19	4–5 /day	2	None	None	None	None
**2**	2	Female	Saga	Rotarix 1^st^ Dose 2013/3/29	2013/3/29-30 2013/4/4	2–3 /day of loose stools	None	None	None	None	None
**3**^a^	22	Male	Saga	None	2013/5/18 2013/5/20	2–4 /day of watery stool	1	38.0	Anemia AGE	Old brother (3 year-old)	None
**4**	3	Male	Osaka	Rotarix 1^st^ Dose 2013/4/2	2013/4/3 2013/4/3	Increased episodes of defecation	None	38.7	None	None	None
**5**	2	Male	Osaka	Rotarix 1^st^ Dose 2013/5/28	2013/6/2 2013/6/3	Increased episodes of defecation	None	None	None	None	None
**6**	5	Female	Osaka	Rotarix 2^nd^ Dose (Unknown)	2014/7/12 2014/7/14	8 in 2 days	None	None	(Unknown)	Old sister	Yes
**7**	4	Male	Osaka	Rotarix 2^nd^ Dose 2014/12/13	2014/12/25 2014/12/27	2–4 /days of loose or watery stool	1	None	(Unknown)	(Unknown)	Yes

^a^ Sequencing of four representative genes demonstrated the presence of G9P[8] rotavirus wild-type strain in patient No.3, indicating false positivity of Rotarix NSP2 assay.

### Viral titration and sanger sequencing of positive samples in Rotarix NSP2 assay

The results of viral titration and sequencing of these seven positive samples are shown in Table 2. Sequencing of four genes (VP7, VP4, VP6, and NSP2) by conventional RT-PCR identified Rotarix-derived strains in four samples (sample No.2, 4, 5 and 7), and both Rotarix-derived strains and rotavirus wild-type strains in two (sample No.1 and 6).

**Table 2:** Results of viral titration and sanger sequencing of four genes of seven positive samples in Rotarix NSP2 assay.

Sample No.	Viral Titer of Rotarix strain (log10 copies/g of stool)	Nucleotide identity with Rotarix original strain (%)				Genotype constellation	Concurrent infection	
		VP7 (1062 bp ^a^/935 bp ^b^)	VP4 (876 bp ^a^/798 bp ^b^)	VP6(1356 bp ^a^/1194 bp ^b^)	NSP2(1058 bp ^a^/946 bp ^b^)		Pathogen	Viral Titer (log10 copies/g of stool)
**1**	11.38	94.4	90.4	80.3 100 (341 bp)	82.3 100 (281 bp)	G1-P[8]-I1/I2-N1/N2	Norovirus GII.4 (2012)	4.62
**2**	9.14	100	100	100	100	G1-P[8]-I1-N1	None	—
**3**	5.45	75.3	90.7	89.1	90.2	G9-P[8]-I1-N1	None	—
**4**	9.10	100	100	100	100	G1-P[8]-I1-N1	None	—
**5**	9.70	100	100	100	100	G1-P[8]-I1-N1	*E*. *coli* O6	—
**6**	5.89	94.4	90.9	90.8	90.0 100 (281 bp)	G1-P[8]-I1-N1	Enterovirus	6.33
**7**	5.51	99.8	99.7	99.9	100	G1-P[8]-I1-N1	Norovirus GII.3	10.11

^a^ Expected length of amplicon.

^b^ Obtained length of amplicon for comparison and calculation of nucleotide identity with Rotarix original strain.

For sample No.1, VP7 (1051 bp), VP4 (803 bp), VP6 (1237 bp) and NSP2 (1030 bp) genes were highly similar (99.9–100% identity) with those of DS-1-like G1P[8] wild-type strain (HC12016) detected in Osaka in 2012 [34]. Along with those wild-type genes, sequences of VP6 (341 bp) and NSP2 (281 bp) which were 100% identical with those of Rotarix original strain were also obtained by conventional RT-PCR. The viral titer of rotavirus wild-type strain was 13.08 log 10 copies/g of stool.

For sample No.6, VP7 (1014 bp), VP4 (811 bp), VP6 (339 bp) and NSP2 (953 bp) genes were highly similar (95.9–99.9% identity) with Wa-like G1P[8] wild-type strain (CK00110) detected in 2011 in Victoria, Australia [35]. Along with those wild-type genes, sequence of NSP2 (281 bp) which was 100% identical with that of Rotarix original strain was also obtained. The viral titer of rotavirus wild-type stain could not be calculated probably due to its low viral titer.

For sample No.3, VP7 (1018 bp), VP4 (810 bp), VP6 (1305 bp) and NSP2 (950 bp) genes were highly similar (99.5–99.9% identity) with those of rotavirus wild-type strains and they had genome constellation of G9-P[8]-I1-N1. Based on the results of sequencing on four genes, the results of Rotarix NSP2 assay was considered false positive.

For two samples (No.1 and No.6) that possessed both Rotarix-derived strains and rotavirus wild-type strains, analyses by NGS were carried out to characterize the viruses that concurrently existed in the intestine. Four out of seven patients (No.1, No.5, No.6 and No.7) had concurrent infection with other pathogens such as norovirus, *E*.*coli* and enterovirus based on the results of multiplex RT-PCR and stool culture. The viral titers of these viruses are also described in Table 2.

### Analysis by NGS

**Fig 1:**
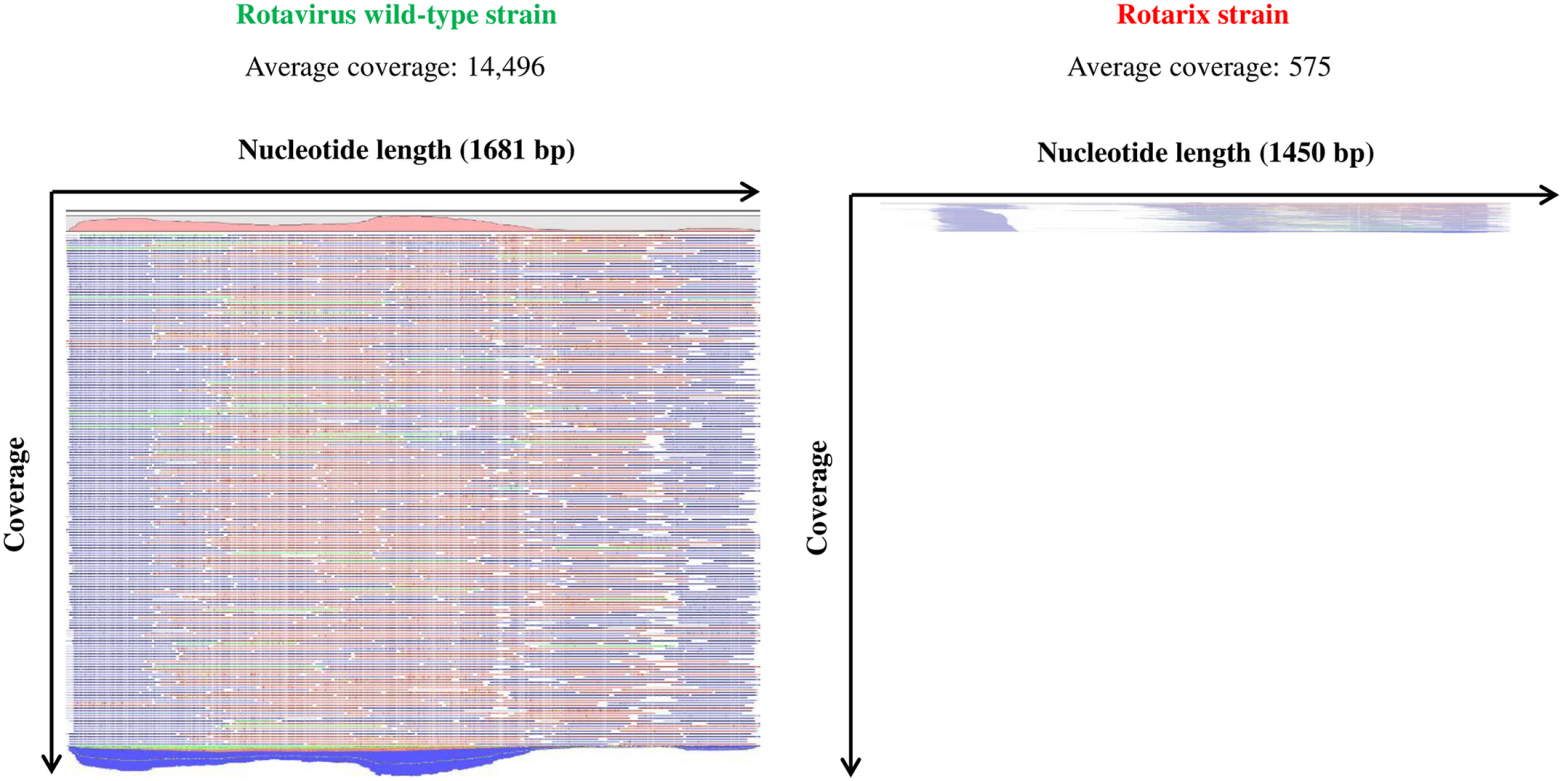
Maps of short reads to cover each contig of VP6 gene in sample No.1. Contig of rotavirus wild-type strain was apparently covered with a larger number of short reads than that of Rotarix strain in sample No.1.

**Fig 2:**
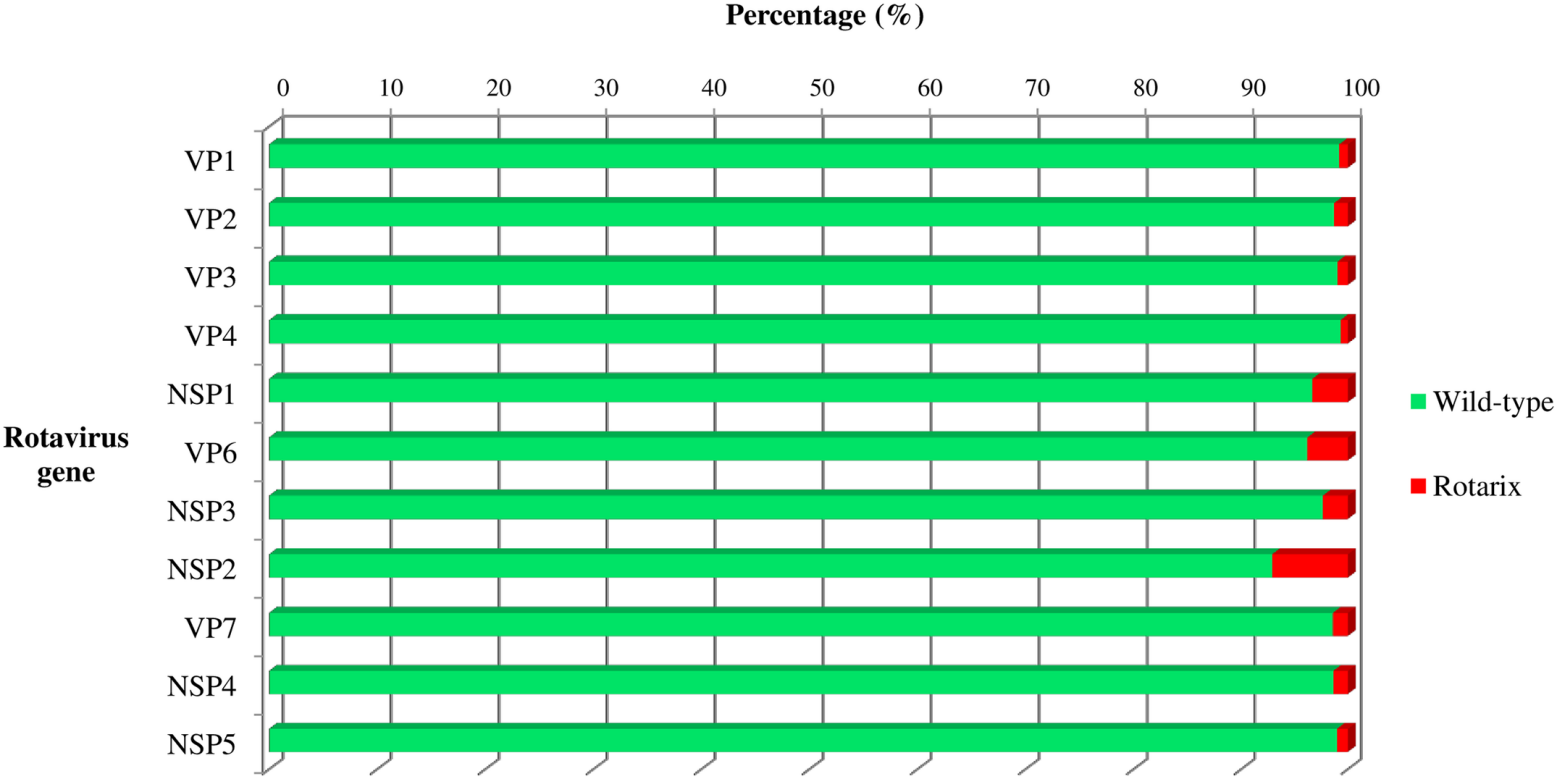
Percentage of wild-type strain and Rotarix strain in sample No.1. The percentage of wild-type strain (green) and Rotarix vaccine strain (red) was calculated for each segmented gene based on the number of average coverage of each contig.

In sample No.1, 8,786 contigs were constructed by CLC genomics workbench and 24 contigs by VirusTap. All the 11 genes of both rotavirus wild-type strain and Rotarix strain were finally identified by BLAST search. Fig 1 shows the maps of short reads to cover each contig of VP6 gene, as a representative example. The number of reads to construct one contig is apparently much larger in wild-type strain than in Rotarix strain. The number of average coverage for each contig is an indicator to show the average times that each nucleotide is sequenced [36]. Accordingly, we calculated the percentage of wild-type strain and Rotarix strain for each gene based on the number of average coverage. As a result, rotavirus wild-type strain existed with higher percentage (92.9–99.3%) compared to Rotarix strain (0.7–7.1%) in all the 11 genes as shown in Fig 2.

Nearly whole nucleotide sequences of all the 11 genes of wild-type strain were obtained in sample No.1. According to the results of genotyping and BLAST search, the genome constellation of the wild-type strain was G1-P[8]-I2-R2-C2-M2-A2-N2-T2-E2-H2, which represented a DS-1-like constellation. In addition, all the genes were highly similar (99.8–100% identity) with those of DS-1-like G1P[8] strain detected in Osaka in 2012 (HC12016, accession nos. AB848004-AB848014) [34].

In sample No.6, 2,500 contigs were constructed by CLC genomics workbench and 10 contigs by VirusTap. Based on the result of BLAST search, contigs derived from rotaviruses could not be identified. Coxsackievirus B4 (CB4), one of the members of genus *Enterovirus* within the family *Picornaviridae*, was detected among contigs constructed by both CLC genomic workbench and Virus Tap.

## Discussion

This study was conducted right after the two rotavirus vaccines had been introduced as voluntary vaccination in Japan. With the vaccine coverage across the country being approximately 50% during the study period, we detected six out of 372 (1.6%) RVA cases of AGE which possessed rotavirus vaccine-derived strains. This is relatively low prevalence compared to the one in a previous study in the US where they reported 4.7% of such cases with the vaccine coverage being around 60% [18,37].

In patient No.2 and No.4, the obtained sequences of VP7, VP4, VP6 and NSP2 gene were 100% identical with those of Rotarix original strain. No other viral or bacterial pathogen was detected from these patients. They had developed mild symptoms of AGE within six days after being vaccinated with 1^st^ dose of Rotarix. As neither of them had siblings nor had attended a daycare, we considered that Rotarix-derived strains in these two patients were vaccines shed after the vaccination, rather than horizontal transmission event. Since we were not able to rule out non-infectious diarrhea, such as intolerances, food allergies, and secondary to the use of unrecorded medicines, the contribution of the vaccine strains to these AGE patients’ symptoms remained unclear.

For patient No.5, we suspected that the main cause of AGE was *E*.*coli* O6, based on the clinical symptom of bloody stool, a finding suggestive of bacterial rather than viral AGE. In a similar manner, the AGE case of patient No.7 probably had been caused by norovirus GII.3 based on the results of real-time RT-PCR which showed viral titer of norovirus was high while that of Rotarix-derived strain was low [38].

In patient No.1 and No.6 from whom both the Rotarix-derived strains and rotavirus wild-type strains were detected, we firstly performed electropherotype profiling by PAGE to confirm the concurrent infection of the two strains and to capture any signs of rearrangement or reassortment in vain (S1 Text). Next, we carried out NGS analyses aiming to characterize the viruses that concomitantly existed in the intestine. In sample No.1, rotavirus wild-type strain accounted for more than 92.9% in all 11 genes. We interpreted NGS data as strongly suggestive of concurrent infection of vaccine-derived strain and the wild-type strains without clear evidence of reassortant strains. Attenuated rotaviruses (such as Rotarix strain) are shed at much lower levels than wild-type strains, and so would the re-assorted strain. Therefore, the higher prevalence of the wild-type rotavirus genomic segments versus vaccine or potential reassortant strain should be expected. Only virus isolation via liquid limited dilutions followed by complete genomic sequencing could distinguish concurrent infection versus reassortment event. In our case, however, this strategy was hindered due to low amount of remaining sample volume. Though norovirus was detected as well, the viral titer was quite low while those of rotavirus wild-type strain (DS-1-like G1P[8]) and Rotarix strain were high. Based on these analyses, we inferred that rotavirus wild-type strain was the probable pathogen of AGE in sample No.1. The infant might have got infected with the wild-type strain soon after he was vaccinated with 1^st^ dose of Rotarix and right before the immune response against rotavirus was established. The Rotarix strain detected in this sample was likely the shed vaccine strain rather than the strain from any horizontal transmission event.

In sample No.6, contigs of rotavirus wild-type strain and Rotarix-derived strain were not successfully constructed. A previous study has demonstrated that the sensitivity and quantitative performance of the NGS-based approach for detection of DNA viral sequences was equivalent to that of real-time PCR in cell-free clinical samples with a sequence depth of 5,000,000 reads [39]. Although Rotarix strain in sample No.6 was quantified as 5.89 log 10 copies/g of stool by real-time RT-PCR, instability of RNA virus and impurities contained in clinical stool sample might have affected the analytical sensitivity of NGS. On the other hand, the result of viral titration by real-time RT-PCR for enterovirus and analyses by NGS both demonstrated the predominance of CB4. Therefore, we inferred that CB4 was the probable pathogen of AGE in this sample.

Of note, among all six AGE cases which possessed Rotarix-derived strains, four (sample No.1, 5, 6 and 7) were suspected to be caused by other pathogens. Most likely, the infants were infected with other pathogens during the shedding period of Rotarix strain. This result exhibited a complex relationship between vaccine strains and clinical illnesses. To properly assess the potential risks of the vaccines, clinical illnesses associated with vaccine or vaccine-derived strains should be evaluated carefully by considering vaccination history and testing other viral or bacterial pathogens.

One false-positive sample (sample No.3) was detected in real-time RT-PCR. One of the reasons to explain this error would be the similarity of nucleotide sequences in both primer and probe binding sites. In order to improve the specificity of detecting vaccine strains, secondary screening assay of real-time RT-PCR, such as Rotarix VP4 assay, or genome sequencing is needed [25].

Although the generalizability of the findings in the current study is limited due to the confined surveillance sites, to the best of our knowledge, this is the first study to assess the prevalence of vaccine-derived strains in patients with AGE in Japan. RotaTeq strains were not detected in this study partly because the vaccine coverage of RotaTeq was lower than that of Rotarix during the study period. However, the AGE cases caused by direct vaccination or horizontal transmission of RotaTeq strains or its reassortants have been reported in other countries [18,19,40]. As the coverage of rotavirus vaccines has increased in Japan, investigation on both Rotarix and RotaTeq strains among AGE patients should be continued to monitor any horizontal transmission case or reassortment event associated with vaccine strains. By monitoring the prevalence of vaccine strains or vaccine-derived strains, we could assess the potential for sustained circulation of these strains.

## Conclusion

The prevalence of vaccine-derived strains in patients with RVA AGE was 1.6% in Japan in 2012-2015. Neither horizontal transmissions nor reassortment event of vaccine strains were observed. The results emphasized the importance of continuous monitoring on vaccine strains and their clinical impacts on children.

## Supporting information

S1 Table Primers and probes used in real-time RT-PCR assays. (PDF)

S2 Table Primers used in conventional RT-PCR and sanger sequencing. (PDF)

S3 Table GenBank accession numbers for representative gene sequences of seven positive samples in Rotarix NSP2 assay. (PDF)

S1 Text Supplementary material for PAGE. (PDF)
