# Supplementary material for: Identification of vaccine-derived rotavirus strains in children with acute gastroenteritis in Japan, 2012-2015
Source: PLoS One. 2017 Sep 13;12(9):e0184067. doi: 10.1371/journal.pone.0184067 (PMC5597190; doi:10.1371/journal.pone.0184067)
Supplement: S1 Table — (PDF) [file pone.0184067.s001.pdf]

S1 Table. Primers and probes used in real-time RT-PCR assays.

Assay	Primer/ Probe	Polarity[a]	Sequence (5'-3')[b]	Nucleotide Position[c] (nt)	Amplicon Size (bp)	Reference
**Rotarix NSP2**	NSP2-F	+	GAACTTCCTTGAATATAAGATCACACTGA	592-620	281	[1]
	NSP2-R	-	TTGAAGACGTAAATGCATACCAATTC	872-847		
	NSP2-P	-	FAM-TCCAATAGATTGAAGT{C}AGTAA“C”GTTTCCA-BHQ1	828-799		
**RotaTeq VP6**	VP6-F	+	GCGGCGTTATTTCCAAATGCACAG	936-959	100	[1]
	VP6-R	-	CGTCGGCAAGCACTGATTCACAAA	1035-1012		
	VP6-P	+	FAM-ATCACGCAA“C”AGTAGGACT“C”ACGCTT-BHQ1	970-995		
**Rotavirus NSP3**	JVKF	+	CAGTGGTTGATGCTCAAGATGGA	17-39	131	[2]
	JVKR	-	TCATTGTAATCATATTGAATACCCA	147-123		
	JVKP	-	FAM-ACAACTGCAGCTTCAAAAGAAGWGT-BHQ	96-72		

[a] +, Forward; -, Reverse.

[b] {C}, AP-dC (G-clamp); “C”, C-5 propynyl-dC (pDC)

[c] The positions correspond toNSP2 (FJ423152), VP6 (FJ423150) and NSP3 (FJ423152) of reference Wa strain.

1. Gautam R, Esona MD, Mijatovic-Rustempasic S, Ian Tam K, Gentsch JR, Bowen MD. Real-time RT-PCR assays to differentiate wild-type group A rotavirus strains from Rotarix(®) and RotaTeq(®) vaccine strains in stool samples. Hum Vaccin Immunother. 2014;10(3):767–77.
2. Jothikumar N, Kang G, Hill VR. Broadly reactive TaqMan assay for real-time RT-PCR detection of rotavirus in clinical and environmental samples. J Virol Methods. 2009;155(2):126–31.
