# Supplementary material for: Identification of vaccine-derived rotavirus strains in children with acute gastroenteritis in Japan, 2012-2015
Source: PLoS One. 2017 Sep 13;12(9):e0184067. doi: 10.1371/journal.pone.0184067 (PMC5597190; doi:10.1371/journal.pone.0184067)
Supplement: S2 Table — (PDF) [file pone.0184067.s002.pdf]

S2 Table. Primers used in conventional RT-PCR and Sanger sequencing.

Gene	Primer	Polarity[a]	Sequence (5'-3')	Nucleotide Position[b] (nt)	Reference
**VP7**	Beg9	+	GGCTTTAAAAGAGAGAATTTCCGTCTGG	1-28	[1]
	End9	-	GGTCACATCATACAATTCTAATCTAAG	1062-1036	[1]
	Mid1r	-	ATTCCTAACGTTTGTGTATT	662-643	[2]
	Mid2	+	GATTTGATATTGAATGAATGG	517-537	[2]
	VP7-1`F	+	AAAGGATGGCCAACAGGATCAGT	373-395	[3]
**VP4**	con3	+	TGGCTTCGCTCATTTATAGACA	11-32	[4]
	con2	-	ATTTCGGACCATTTATAACC	887-868	[4]
**VP6**	VP6-F	+	GGCTTTTAAACGAAGTCTTC	1-20	[5]
	VP6-R	-	GGTCACATCCTCTCACT	1356-1339	[5]
**NSP2**	NSP2-F	+	GGCTTTTAAAGCGTCTCAGTC	1-21	[6]
	NSP2-R	-	GGTCACATAAGCGCTTTCTATTC	1058-1036	[6]

[a] +, Forward; -, Reverse.

[b] The positions correspond to VP7 (K02033), VP4 (FJ423116), VP6 (FJ423150) , NSP2 (FJ423152) of reference Wa strain.

1. Gouvea V, Glass RI, Woods P, Taniguchi K, Clark HF, Forrester B, et al. Polymerase chain reaction amplification and typing of rotavirus nucleic acid from stool specimens. J Clin Microbiol. 1990;28(2):276–82.
2. Trinh QD, Nguyen TA, Phan TG, Khamrin P, Yan H, Le Hoang P, et al. Sequence analysis of the VP7 gene of human rotavirus G1 isolated in Japan, China, Thailand, and Vietnam in the context of changing distribution of rotavirus G-types. J Med Virol. 2007;79(7):1009–16.
3. Yan H, Nguyen TA, Phan TG, Okitsu S, Li Y, Ushijima H. Development of RT-multiplex PCR assay for detection of adenovirus and group A and C rotaviruses in diarrheal fecal specimens from children in China. 2004;78(8):699–709.
4. Gentsch JR, Glass RI, Woods P, Gouvea V, Gorziglia M, Flores J, et al. Identification of group A rotavirus gene 4 types by polymerase chain reaction. J Clin Microbiol. 1992 ;30(6):1365–73.
5. Shen S, Burke B, Desselberger U. Rearrangement of the VP6 gene of a group A rotavirus in combination with a point mutation affecting trimer stability. J Virol. 1994 ;68(3):1682–8.
6. Fujii Y, Shimoike T, Takagi H, Murakami K, Todaka-Takai R, Park Y, et al. Amplification of all 11 RNA segments of group A rotaviruses based on reverse transcription polymerase chain reaction. Microbiol Immunol. 2012;56(9):630–8.
