# Supplementary material for: Identification of vaccine-derived rotavirus strains in children with acute gastroenteritis in Japan, 2012-2015
Source: PLoS One. 2017 Sep 13;12(9):e0184067. doi: 10.1371/journal.pone.0184067 (PMC5597190; doi:10.1371/journal.pone.0184067)
Supplement: S3 Table — (PDF) [file pone.0184067.s003.pdf]

S3 Table. GenBank accession numbers for representative gene sequences of seven positive samples in Rotarix NSP2 assay.

削除: nome

Sample No.	GenBank accession numbers										
	VP1	VP2	VP3	VP4	NSP1	VP6	NSP3	NSP2	VP7	NSP4	NSP5
1[a]	KY616885	KY616886	KY616887	KY616888	KY616889	KY616890	KY616891	KY616892	KY616893	KY616894	KY616895
2	—	—	—	—	—	—	—	—	—	—	—
3	—	—	—	—	—	—	—	—	—	—	—
4	—	—	—	KY616896	—	KY616897	—	KY616898	KY616899	—	—
5	—	—	—	—	—	—	—	—	—	—	—
6	—	—	—	KY616900	—	KY616901	—	KY616902	KY616903	—	—
7	—	—	—	KY616904	—	KY616905	—	KY616906	KY616907	—	—

[a] Only genome sequences of RVA wild-type strains were deposited.
