# Supplementary material for: Identification of vaccine-derived rotavirus strains in children with acute gastroenteritis in Japan, 2012-2015
Source: PLoS One. 2017 Sep 13;12(9):e0184067. doi: 10.1371/journal.pone.0184067 (PMC5597190; doi:10.1371/journal.pone.0184067)
Supplement: S1 Text — (PDF) [file pone.0184067.s004.pdf]

S1 Text. Supplementary Material for PAGE

RNA PAGE has been widely utilized to characterize new rotavirus strains found in epidemiological studies as the dsRNA migration pattern can be directly visualized with relatively simple equipment [1]. According to previous studies, for samples of concurrent infection with several rotavirus strains, they presented with more than 11 RVA segments on PAGE [2]. For samples possessing viruses with rearrangement, typical RNA segments are missing or are decreased in concentration and replaced by additional, more slowly migrating bands of RNA [3,4]. Since Rotarix original strain had a long electropherotype pattern which was similar but not identical with that of Wa-like strain [5], we hypothesized that the pattern of wild-type strains and Rotarix strain could be distinguished to show either reassortment or concurrent infection happened in sample No.1 and No.6. As a result, those samples that had low viral titers approximately under 12.0 log 10 copies/g of stool were unlikely to show any electropherotype patterns. In sample No.1, only wild-type strain, which had viral titer of 13.08 log 10 copies/g of stool, showed a short electropherotype pattern of DS-1-like strain. Sample No.6, which had low viral titer of both wild-type strain and Rotarix-derived strain, yielded no expected pattern. In summary, it was impossible to apply the results of PAGE to interpret the existence of two strains in both samples.

1. Herring AJ, Inglis NF, Ojeh CK, Snodgrass DR, Menzies JD. Rapid diagnosis of rotavirus infection by direct detection of viral nucleic acid in silver-stained polyacrylamide gels. J Clin Microbiol. 1982;16(3):473-7.
2. Nyaga MM, Jere KC, Esona MD, Seheri ML, Stucker KM, Halpin RA, et al. Whole genome detection of rotavirus mixed infections in human, porcine and bovine samples co-infected with various rotavirus strains collected from sub-Saharan Africa. Infect Genet Evol. 2015;31:321–34.
3. Estes MK, Greenberg HB. Rotaviruses. In: Kinpe DM, Howley PM, editors. Fields virology sixth edition. Philadelphia: Lippincott; 2013.1347–401.
4. Schnepf N, Deback C, Dehee A, Gault E, Parez N, Garbarg-Chenon A. Rearrangements of rotavirus genomic segment 11 are generated during acute infection of immunocompetent children and do not occur at random. J Virol. 2008;82(7):3689–96.
5. Nakagomi T, Nakagomi O, Dove W, Doan YH, Witte D, Ngwira B, et al. Molecular characterization of rotavirus strains detected during a clinical trial of a human rotavirus vaccine in Blantyre, Malawi. Vaccine. 2012;30 Suppl 1(01):A140-51.
